# Early pain relief and segmental realignment after two-stage lateral lumbar interbody fusion (LLIF) with posterior instrumentation in degenerative lumbar pathology

**DOI:** 10.1007/s10143-026-04351-5

**Published:** 2026-06-04

**Authors:** Meltem Ivren, Basem Ishak, Sebastian Ille, Martin Dugas, Sandro M Krieg, Pavlina Lenga

**Affiliations:** 1 Department of Neurosurgery, Heidelberg University Hospital, Im Neuenheimer Feld 400, Heidelberg, 69120 Germany; 2 Department of Neurosurgery, ATOS Klinik Wiesbaden, Wiesbaden, Germany; 3 Institute of Medical Informatics, Heidelberg University Hospital, Heidelberg, Germany

**Keywords:** Lateral lumbar interbody fusion (LLIF), Staged circumferential fusion, Degenerative lumbar spine disease, Indirect decompression

## Abstract

Two-stage circumferential fusion incorporating lateral lumbar interbody fusion (LLIF) is increasingly used to treat degenerative lumbar disorders, yet evidence on perioperative safety, clinical benefit, and optimal sequencing remains limited. We evaluated clinical outcomes, radiographic realignment, perioperative morbidity, and predictors of complications in patients undergoing staged LLIF with posterior fixation. We retrospectively reviewed 20 patients (mean age 67.7 years) who underwent two-stage lumbar fusion, either LLIF followed by posterior instrumentation or vice versa. We assessed pain (NRS scale), segmental lordosis (SL), blood loss, ICU and hospital length of stay, and complications. Subgroup analysis compared LLIF-first vs. posterior-first sequencing. Logistic regression identified predictors of complications. Patients showed a median age-adjusted Charlson Comorbidity Index [ACCI] of 3.0. LLIF was performed first in 65%. Mean NRS pain scores improved from 6.5 preoperatively to 4.1 after the first stage (*p* = 0.006) and to 3.1 after the second (*p* < 0.001). Early pain relief tended to be greater with LLIF-first (ΔNRS − 2.46 vs. − 1.75). Posterior-first sequencing involved more instrumented levels (median 4 vs. 2, *p* = 0.014), greater blood loss (628.6 vs. 142.3 mL, *p* = 0.01), and longer hospitalization (18.9 vs. 10.8 days, *p* < 0.001). LLIF-first yielded greater intervertebral height gain (6.5 vs. 3.7 mm, *p* = 0.05). SL improved mainly at upper lumbar levels (+ 6.4°). ACCI was the only independent predictor of complications (OR 2.6 per point; *p* = 0.045). Staged lateral lumbar interbody fusion with posterior instrumentation appears to be a feasible treatment option for complex degenerative lumbar disease, associated with early pain relief and segmental alignment changes. Performing the interbody fusion as the first stage may offer potential advantages; however, these findings should be interpreted cautiously given the small sample size of our cohort.

## Introduction

Low back pain (LBP) remains a leading global contributor to disability, predominantly driven by degenerative lumbar spinal pathology [8]. While conservative therapies are effective as initial interventions, a substantial proportion of patients with degenerative lumbar spine disorders ultimately require surgical stabilization to achieve meaningful symptom relief and functional restoration [13, 17]. Lumbar spinal fusion, particularly circumferential (360°) fusion—which integrates anterior or lateral interbody fusion with posterior instrumentation—is increasingly adopted to optimize biomechanical stability, improve fusion rates, and maintain long-term spinal alignment [19]. However, performing circumferential fusion in a single surgical setting often poses significant logistical and physiological challenges, including prolonged anesthesia, extended operative time, intraoperative patient repositioning, and increased perioperative stress [14, 19]. To overcome these limitations, a staged circumferential fusion approach has been proposed, wherein anterior or lateral interbody fusion and posterior instrumentation are performed sequentially across separate surgical sessions. Previous studies suggest that this two-stage strategy reduces physiological strain, allows interim patient reassessment, optimizes resource utilization, and may potentially decrease intraoperative and perioperative complication rates compared to single-stage surgery [19]. Nevertheless, the current body of literature presents conflicting evidence regarding safety profiles, complication rates, and patient outcomes associated with staged versus single-stage circumferential fusion approaches [14, 19]. Consequently, clinical guidelines on optimal surgical staging in degenerative lumbar spine surgery remain ambiguous [19].

The present study systematically evaluates perioperative safety, radiographic alignment, and patient-reported outcomes following a two-stage 360° lumbar fusion approach involving lateral lumbar interbody fusion (LLIF) and posterior instrumentation. By analyzing clinical outcomes and perioperative safety, our study directly addresses critical gaps in existing knowledge. Ultimately, we aim to provide clinically relevant data to inform surgical decision-making in patients with complex degenerative lumbar spine pathology.

## Materials and methods

### Study design

This retrospective study evaluated data drawn from our institutional database spanning October 2023 to December 2024. We collected information on demographics, comorbidities, imaging parameters, clinical outcomes, and complication rates to analyze treatment results in patients undergoing two-stage spinal surgery. Ethical approval was obtained from our local ethics committee (approval number 880/2021). The research complied with the principles of the Declaration of Helsinki. All patients ≥ 18 years old who underwent two-stage spinal surgery for degenerative lumbar disease within the study period were included.

### Demographic, clinical, and surgical data

We assessed patient age, sex, body mass index (BMI), American Society of Anesthesiologists (ASA) classification, and the age-adjusted Charlson Comorbidity Index (CCI) [6, 7]), to comprehensively characterize patient health status and operative risk. Surgical and anesthesia records were reviewed to obtain details on operative approach (LLIF, or posterior instrumentation), operative duration, intraoperative blood loss (quantified in milliliters), and blood transfusion requirements. Perioperative complications were documented. Postoperative outcomes, including total hospital length of stay, intensive care unit (ICU) duration, and in-hospital and 90-day mortality rates, were systematically recorded in hospital electronic medical records. Preoperative imaging was standardized across the cohort, routinely including magnetic resonance imaging (MRI) of the lumbar spine to assess degenerative changes, disc pathology, neural element compression, and vertebral alignment. In cases of prior spinal instrumentation, clinical suspicion of instability, or ambiguous MRI findings, computed tomography (CT) scans were performed to delineate further bony anatomy and the integrity of existing spinal hardware. Moreover, for patients with spondylolisthesis identified on MRI or CT scans, we obtained preoperative functional X-rays (flexion and extension) regardless of Meyerding grade to evaluate segmental mobility and dynamic instability objectively.

### Radiographic measurements

Segmental lordosis was quantified using the standardized Cobb angle measurement [5]. The lordotic angle was precisely defined as the angle subtended between the superior endplate of the cranial vertebra and the inferior endplate of the caudal vertebra at the treated segments. Segmental lordosis was consistently measured preoperatively and postoperatively using either sagittal reconstructed imaging (CT/MRI) or standing lateral radiographs, depending on image availability and quality. For patients undergoing multi-level fusion procedures, segmental lordosis was individually measured at each surgically treated segment, and the mean of these values was calculated to represent overall segmental lordotic correction achieved by surgery. Postoperative imaging was systematically performed at each surgical stage to track incremental radiographic changes precisely. Segmental malalignment was defined as segmental lordosis values outside physiological ranges for the respective lumbar levels. As no universally accepted cut-offs exist, reference ranges were derived from previously reported segment- specific lordosis values in the literature, which demonstrate increasing lordotic angles from the upper to lower lumbar spine [4, 10]. The following ranges were used for descriptive analysis: upper lumbar (L1–L3): 3–9°, mid-lumbar (L3–L4): 7–12°, and lower lumbar (L4–S1): 10–25°.

### Pain assessment

Clinical outcomes were assessed using the Numeric Rating Scale (NRS). Due to the retrospective study design, pain assessments were not performed at strictly predefined time points but extracted from clinical documentation. For postoperative pain assessment, we did not use values from the first postoperative day, but rather pain scores obtained after initial mobilization. Final postoperative pain scores were recorded mostly the day before hospital discharge. Outcome assessment focused on the early postoperative period during the index hospitalization. No standardized long-term follow-up was available.

### Adverse events

Perioperative complications were routinely documented in a standardized institutional reporting system. Adverse events were routinely assessed and recorded at discharge by the responsible treating physician and subsequently reviewed by a senior attending. All events were entered into a continuously maintained institutional database, which is regularly cross-checked for completeness. In addition, readmissions within 30 days are systematically captured. For the present study, complications occurring within the first 30 postoperative days were included.

### Indications for surgery

All surgical cases included in this study were prospectively reviewed and discussed at a structured multidisciplinary spine conference comprising experienced neurosurgeons and neuroradiologists. Surgical indications were individualized and documented based on radiographic criteria (evidence of instability, severe foraminal or spinal canal stenosis, disc herniation, or adjacent segment disease), patient-reported clinical symptoms (refractory pain, neurologic deficits, impaired mobility), and failure of adequate conservative therapy (physiotherapy, analgesics, epidural injections). Surgical decision-making prioritized restoring neurological function, relieving refractory pain, improving functional mobility, and enhancing patient-reported quality of life.

The choice of surgical sequence (LLIF first vs. posterior instrumentation first) was based on individual clinical and radiographic considerations. In cases with pronounced central stenosis, significant lateral recess compromise, or relevant scar tissue (e.g., in adjacent segment disease), posterior decompression with fixation was preferentially performed as the first stage.

### Statistical analysis

Statistical analyses were performed using SPSS software (version 27, IBM Corp.). The Shapiro-Wilk test was used to assess the normality of the data distribution. Categorical variables are reported as counts and percentages, whereas continuous variables are expressed as means ± standard deviations (SD). Binary logistic regression was applied to identify predictors of perioperative and postoperative complications. Depending on the variable’s normality and measurement scale, differences in central tendency were assessed using the Student’s t-test or the Mann-Whitney U test. Fisher’s exact test was used for crosstab analyses with small sample sizes. Changes in pain scores were analyzed using the Wilcoxon signed-rank test, as data were paired and not assumed to be normally distributed given the small sample size.

## Results

### Demographics and baseline characteristics

The study cohort consisted of 20 patients, evenly distributed between males and females (*n* = 10 each, 50.0%). The patients were predominantly elderly, with a mean age of 67.7 years (± 11.7), and exhibited moderate comorbidity burden, as reflected by a median age-adjusted Charlson Comorbidity Index (ACCI) of 3.0 (range: 0–6). All patients were categorized as ASA class II (45%) or III (55%), highlighting moderate anesthetic risk. The primary surgical site was the lumbar region (90.0%). Spinal canal or neuroforaminal stenosis was the leading surgical indication (60.0%), of which 58% (*n* = 7) were adjacent-segment disease cases. Spondylolisthesis was present in 30%. At baseline, all patients reported significant pain, with a median NRS pain score of 6.5 (range: 4–9). Motor deficits were observed in 4 patients (20.0%). Detailed baseline data are presented in Table 1.

**Table 1:** Baseline characteristics

	*n* = 20
Age, y (mean, SD)	67.7 (11.7)
**Sex (n**,** %)**	
**Male**	10 (50.0)
**Female**	10 (50.0)
**Charlson Comorbidity Index** (median, range)	3.0 (0–6)
**BMI** (mean, SD)	27.6 (11.7)
**ASA class (n,%)**	
I II III IV V	0 (0.0) 9 (45.0) 11 (55.0) 0 (0.0) 0 (0.0)
**Localization (n**,**%)**	
Thoracolumbar Lumbar Lumbosacral	1 (5.0) 18 (90.0) 1 (5.0)
**Disc height [mm]** (mean, SD)	6.9 (4.8)
**Etiology**	
- Spondylolisthesis - Spinal canal/neuroforaminal stenosis - Adjacent segment disease* - Disc herniation	6 (30.0) 5 (25.0) 7 (35.0) 2 (10.0)
**Neurological symptoms**	
Pain Motor Sensory Sphincter/bladder dysfunction Gait ataxia	20 (100.0) 4 (20.0) 5 (25.0) 0 (0.0) 0 (0.0)
**Preoperative pain on the NRS scale (median**,** range)**	6.5 (4–9)

*ASA* = American Society of Anesthesiologists Physical Status classification, *BMI* = Body Mass Index, *NRS* = Numeric Rating Scale, *SD* = Standard Deviation

*Adjacent segment disease, although a subgroup of spinal canal stenosis, was separately mentioned to give a more detailed overview of the cohort

### Surgical procedure and perioperative outcomes

All patients underwent a planned two-stage surgical approach, with an average interval between stages of 4.9 days (± 2.3). Regarding surgical sequence, the LLIF procedure was performed first in the majority of the cases (*n* = 13, 65.0%) and second in seven cases (35%). One LLIF cage was implanted in the majority of the cases (*n* = 16, 80.0%), two LLIF cages in four cases (20.0%). While there was no significant difference in the number of LLIF levels depending on the surgical sequence, levels of dorsal pedicle screw fixation were significantly longer in cases where the LLIF procedure followed dorsal fixation (median number of levels 4.0 vs. 2.0, *p* = 0.014) (comp. Table 2). Accordingly, dorsal fixation surgery was significantly longer (LLIF 2nd group: 339.6 min. vs. LLIF first group: 154.1 min., *p* = 0.004) with higher intraoperative blood loss rates (628.6 vs. 142.3 ml, *p* = 0.01). Surgery duration, however, did not differ significantly when calculated per segment.

**Table 2:** Surgical Approaches and Postoperative Outcomes

*n* = 20	LLIF 1st *n* = 13		LLIF 2nd *n* = 7		*p* value
Number of levels LLIF median (range)	1.0 (1–2)		1.0 (1–1)		0.110
Number of levels dorsal fixation median (range)	2.0 (0.8, 1–3)		4.0 (1–5)		**0.014**
Intervertebral space preop. [mm] * mean (SD)	5.5 (2.8)		7.7 (2.8)		0.112
Intervertebral space postop. [mm] * mean (SD)	12.1 (2.5)		11.4 (5.2)		0.706
Δ Intervertebral space mean (SD)	6.5 (2.3)		3.7 (3.8)		0.05
Duration of LLIF surgery (min) (mean, SD)	89. 2 (43.1)		111.5 (57.3)		0. 358
*Duration LLIF per segment* *(mean*,* SD)*	67.9 (21.8)		111.5 (57.3)		0.114
Duration of dorsal fixation surgery (min) (mean, SD)	153.1 (25.440)		339.6 (109.2)		**0.004**
*Duration dorsal fixation per segment* *(mean*,* SD)*	104.7 (49.8)		121.4 (71.1)		0.711
*Intraoperative blood loss [ml] (mean, SD)*					
- LLIF-procedure - Dorsal fixation procedure	69.2 (149.4) 142. 3 (163.1)		264.3 (402.8) 628.6 (460.8)		0.149 **0.01**
*Intraoperative blood transfusion (n*,* %)*					
- LLIF-procedure - Dorsal fixation procedure	1 (7.7) 0 (0.0)		0 (0.0) 2 (28.7)		0.650 0.614
Days between surgeries (mean, SD)	4.9 (2.3)				
Hospital stay (mean, SD)					
*Overall*		*13.6 (5.3)*			
*Per surgical sequence group*	10.8 (3.4)	18.9 (4.0)		***p *** **< 0.001**	
ICU stay (mean, SD)	0.4 (0.5)				
Mortality *(n*,* %)*					
- In hospital − 90 days	0 (0.0) 0 (0.0)				
**Complication rate** (*n* = 5,** 25%)(n**,** %)**	2/13 (15.4)	3/7 (42.9)		***p*** =0.290	
**Surgery-related **(*n* = 4,** 20%)**	1 (7.7)	3 (42.9)			
- Impaired wound healing		1 (14.3)			
- Cage dislocation		1 (14.3)			
- New permanent paresis	1 (7.7)				
- Psoas muscle injury		1 (14.3)			
**Other **(*n* = 1,** 5%)**					
- Acute kidney failure	1 (7.7)				

*ICU* = Intensive Care Unit, preop. = before LLIF, postop. = after LLIF, min = minutes, Δ Intervertebral space = Difference of intervertebral space after - before LLIF surgery, *LLIF* = Lateral Lumbar Interbody Fusion, *SD* = Standard Deviation, *LLIF* *1st* = Lateral Lumbar Interbody Fusion (LLIF) was performed as first surgery before dorsal fixation, *LLIF 2nd* = Lateral Lumbar Interbody Fusion (LLIF) was performed as second surgery after dorsal fixation

The intervertebral space as a marker of indirect decompression was more feasible when LLIF was performed before pedicle screw fixation (mean difference of 6.5 mm vs. 3.7 mm, *p* = 0.05). The average hospital stay was 13.6 days (± 5.3), with a brief mean ICU stay of 0.4 days (± 0.5), primarily for safe extubation following prone surgery. No patients required ICU care beyond one day, and no mortalities occurred (comp. Table 2). Patients who received dorsal instrumentation with decompression first, and LLIF in the second stage, had significantly more extended hospitalization (mean length of stays 18.9 days vs. 10.8 days, *p* < 0.001, comp. Table 2).

The overall complication rate was 25.0%. Surgery related complications, including cage dislocation and new neurological deficits, were rare (5.0% each). Binary logistic regression identified the ACCI as the only significant predictor of complications (OR 2.6; *p* = 0.045), indicating a 164% increased risk of complications for each incremental ACCI point. No significant associations were found with age, sex, BMI, smoking status, neurological deficits, ASA classification, number of surgical levels, surgical duration, or blood loss.

### Segmental lordosis correction and clinical outcomes

Postoperative radiographic evaluation demonstrated a numerical increase in segmental lordosis (SL) across lumbar segments, most notably at the upper lumbar levels (L1–L3), where mean SL increased from 2.4° (± 9.6) preoperatively to 8.8° (± 3.0) postoperatively, corresponding to a mean gain of 6.4°.

**Fig. 1:**
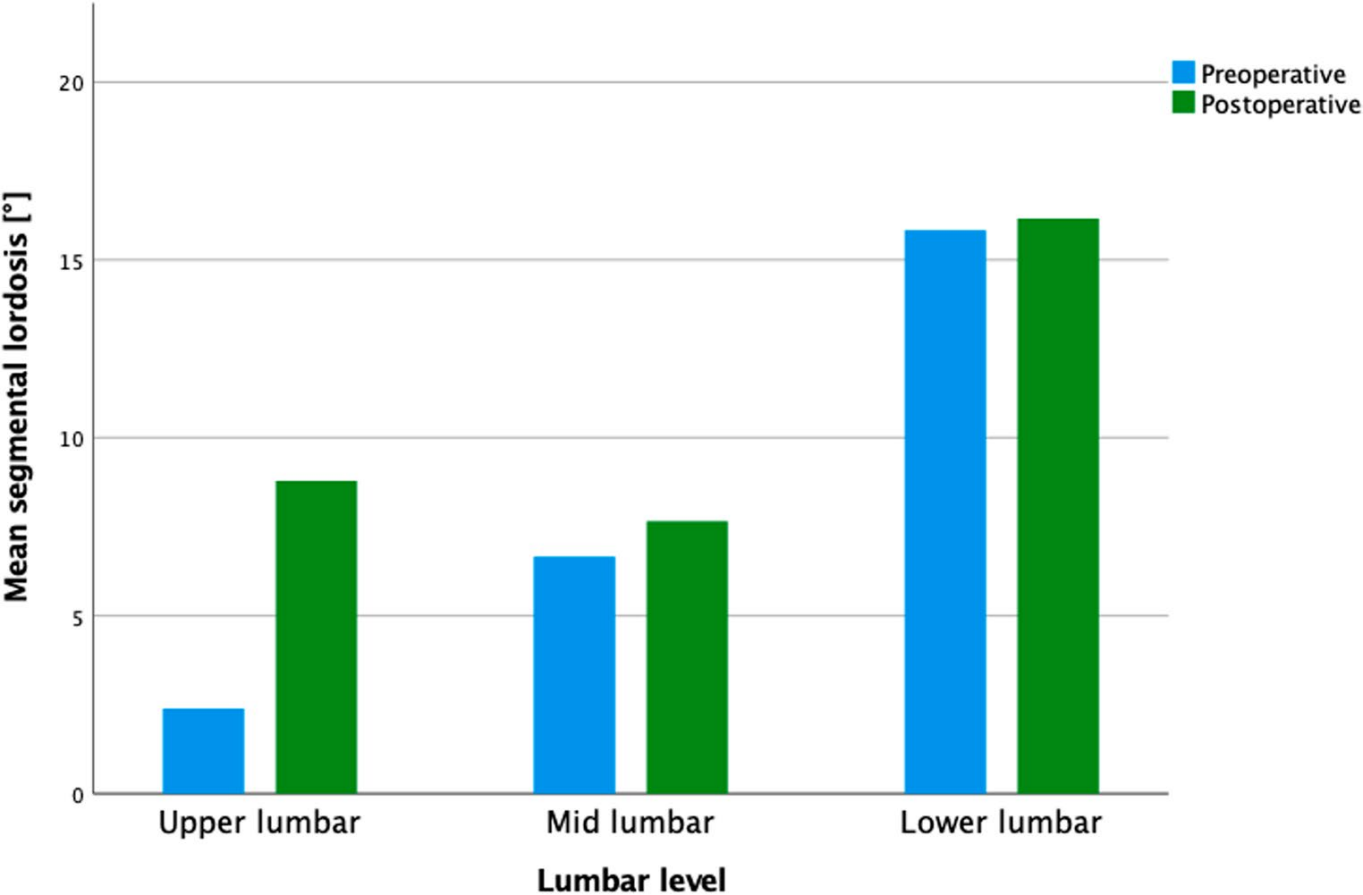
Segmental lordosis before and after staged surgery, stratified by lumbar level. The most pronounced postoperative gain was observed in the upper lumbar segment (L1–L3), with more modest increases in the mid lumbar region (L3–L4) and minimal change in the lower lumbar segment (L4–S1)

Mid-lumbar (L3–L4) segments showed modest improvement from 6.7° to 7.7° (Δ = 1.0°), while lower lumbar segments (L4–S1) showed negligible postoperative change (15.8° ± 7.5 to 16.2° ± 8.8; Δ = 0.33°). Results of these changes are summarized in Fig. 1. Subgroup analysis based on surgical sequence revealed distinct differences in segmental lordosis outcomes. In the total cohort, the rate of unphysiological lordosis in the upper lumbar region significantly decreased from 60% preoperatively to 20% postoperatively. Within the mid-lumbar segment, an increase in the proportion of segments classified as mid-lumbar malalignment (44.4% preoperative to 100% postoperative) was observed. Lower lumbar alignment remained unchanged at 33.3% malalignment (Table 3). 

**Fig. 2:**
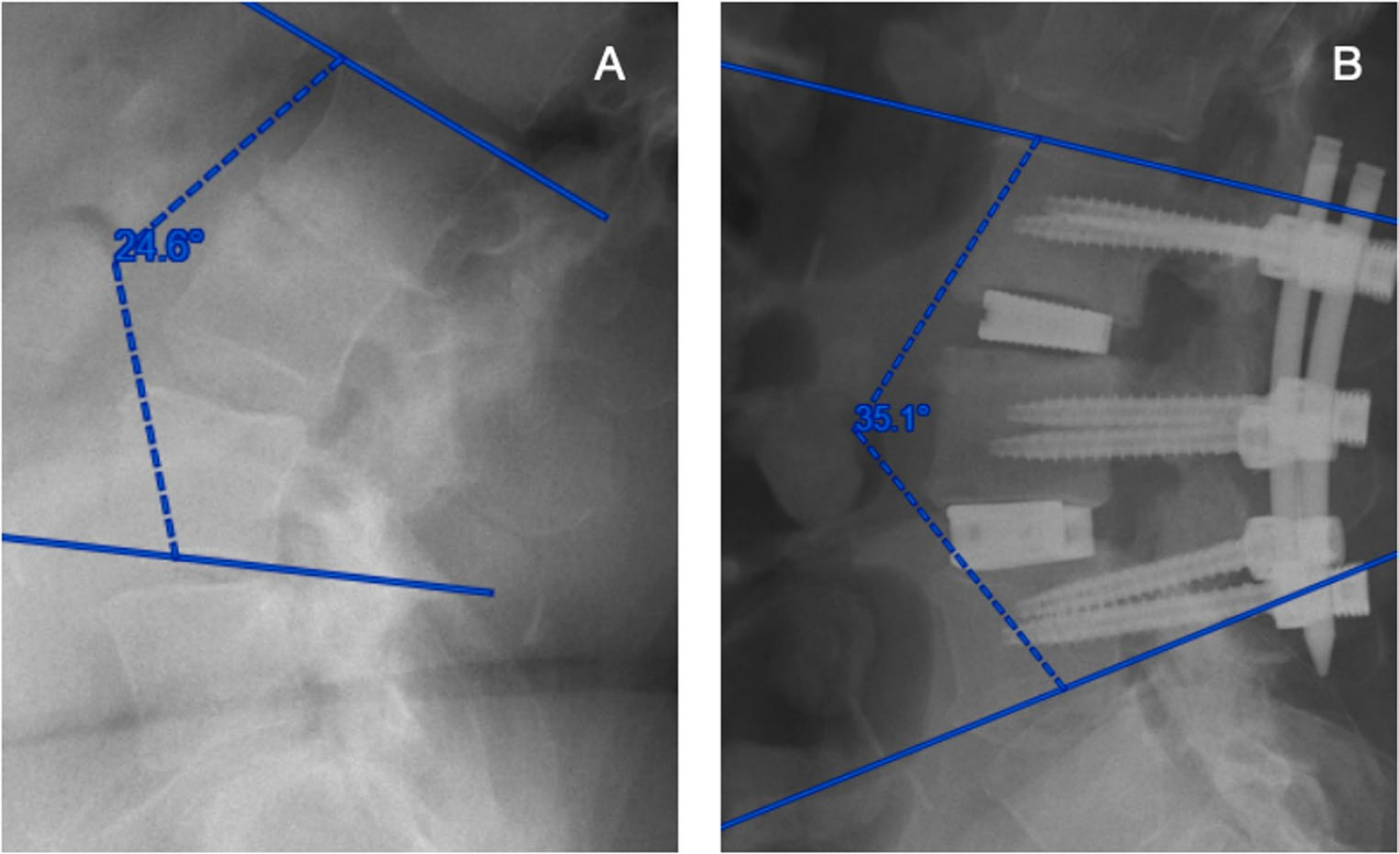
Illustrative case of a two-level LLIF with posterior instrumentation. Pre- (**A**) and postoperative (**B**) X-rays of a 56-year-old female patient with therapy-resistant back pain radiating to the right leg. She had previously undergone a right hemilaminectomy of L4. However, multiple degenerative changes of the spine persisted, including multisegmental recess stenoses, partially worn-out disc spaces, and left-convex scoliosis. The patient underwent a two-stage procedure consisting of a two-level LLIF at L2/3 and L3/4, followed by posterior instrumentation. Lordosis correction across both segments shows a gain of approximately 10°. The patient’s pain improved from 7 to 3 on the NRS scale

**Table 3:** Correction of Segmental Lordosis (overall cohort)

Patients with unphysiological SL*	Preoperatively	Postoperatively**
**Overall cohort **(*n* = 20)		
Upper lumbar (*n* = 4)	3/5 = 60%	1/5 = 20%
Mid lumbar (*n* = 0)	4/9 = 44.4%	9/9 = 100%
Lower lumbar (*n* = 3)	2/6 = 33.3%	2/6 = 33.3%
**LLIF 1 st **(*n* = 13)		
Upper lumbar (*n* = 1)	1/1 = 100%	0/1 = 0.0%
Mid lumbar (*n* = 9)	4/9 = 44.4%	9/9 = 100.0%
Lower lumbar (*n* = 3)	1/3 = 33.3%	1/3 = 33.3%
**LLIF 2nd **(*n* = 7)		
Upper lumbar (*n* = 4)	2/4 = 50%	1/4 = 25%
Mid lumbar (*n* = 0)	0/0	0/0
Lower lumbar (*n* = 3)	1/3 = 33.3%	1/3 = 33.3%

* The following physiological ranges were taken as reference: Upper lumbar (L1-L3): 3–9°; Mid lumbar (L3-L4): 7–12°, Lower lumbar (L4-S1): 10–25°

** Postoperatively after second surgery

LLIF 1 st = Lateral Lumbar Interbody Fusion (LLIF) was performed as first surgery before dorsal fixation

LLIF 2nd = Lateral Lumbar Interbody Fusion (LLIF) was performed as second surgery after dorsal fixation

When comparing surgical sequences specifically, patients undergoing posterior fixation first (*n* = 7) showed improved upper lumbar lordosis (malalignment reduced from 50% to 25%), while lower lumbar lordosis remained unchanged. No mid-lumbar segments were treated in this subgroup. Conversely, patients undergoing LLIF first (*n* = 13) achieved complete normalization of upper lumbar alignment (from 100% to 0% malalignment), yet experienced an unexpected increase in mid-lumbar malalignment postoperatively (44.4% to 100%).

Clinically, pain scores improved progressively throughout staged treatment. Mean baseline NRS pain scores (6.5 ± 1.5) decreased to 4.1 ± 2.3 (*p* = 0.006) following the initial surgical stage (*n* = 17). The LLIF-first subgroup demonstrated a more pronounced immediate pain reduction (ΔNRS − 2.46) compared to the posterior-first group (ΔNRS − 1.75), although this difference did not reach statistical significance. After completing both stages, mean pain scores further decreased to 3.1 ± 2.0 (*p* < 0.001), indicating cumulative clinical benefit. An illustrative case for a 56-year old patient undergoing two-level LLIF, followed by posterior instrumentation, is shown in Fig. 2.

## Discussion

In this study, a staged lumbar fusion approach combining lateral lumbar interbody fusion (LLIF) with posterior instrumentation was associated with early postoperative pain reduction in patients with degenerative lumbar pathology. Pain scores decreased following the first-stage LLIF procedure, consistent with the concept of indirect decompression. Segmental lordosis showed a numerical increase, most notably at upper lumbar levels (L1–L3), whereas changes at mid-lumbar segments were more limited. Complication risk was primarily associated with patient comorbidity (OR 2.6 per additional ACCI point). Overall, these findings suggest that staged LLIF and posterior instrumentation may represent a feasible treatment option for elderly and medically complex patients. However, given the retrospective design and limited sample size, these results should be interpreted with caution and considered hypothesis-generating.

### Early clinical improvement after first-stage LLIF

Systematic reviews, including Cochrane analyses on lumbar fusion for degenerative spinal disease, have demonstrated that lumbar spine fusion provides clinical benefit in selected patients regarding pain and function [15, 16]. However, there is a lack of clear evidence regarding the superiority of specific surgical approaches [16].

A significant finding in this study was the early postoperative pain reduction experienced by patients after the first-stage lateral lumbar interbody fusion (LLIF), before posterior instrumentation. The observed pain reduction between the two surgical stages strongly supports the efficacy of LLIF’s indirect neural decompression. This early benefit can be attributed primarily to the restoration of disc height, foraminal widening, and subsequent alleviation of neural compression facilitated by the lateral cage placement [1]. Our results closely align with previously reported findings; for example, Park et al. demonstrated similarly substantial early pain relief (VAS score reduced from 7.6 to 4.1 within one week) following staged lateral interbody fusion before the addition of posterior screws [12]. Additionally, stand-alone XLIF (extreme lateral interbody fusion) has consistently demonstrated rapid alleviation of radicular symptoms in degenerative foraminal stenosis, further emphasizing the efficacy of indirect decompression achieved through minimally invasive anterior approaches [1]. This effect is also reflected in the widening of intervertebral spaces, which was expectedly greater when LLIF was performed before vs. after dorsal instrumentation (6.5 vs. 3.7 mm, mean difference through LLIF cage, *p* = 0.05). The magnitude of pain reduction observed in our cohort—approximately 40–50% following LLIF—is noteworthy and consistent with existing evidence supporting staged fusion strategies. However, these findings should be interpreted cautiously in the context of our retrospective study design with no standardized timing of pain assessment, no long-term follow-up, and the influence of postoperative analgesic medication.

Nevertheless, early symptomatic improvement is often underappreciated or undocumented in traditional single-stage procedures, where clinical outcomes typically emerge weeks after the full surgical intervention. Our two-stage design highlights the direct contribution of the interbody fusion alone, emphasizing its independent therapeutic value. This finding suggests that carefully selected patients with degenerative pathology, such as neuroforaminal stenosis or severe disc-space collapse, may derive substantial immediate clinical benefit from LLIF alone, potentially guiding surgical decision-making and staging strategies. However, in 7 cases (35%), LLIF followed prior dorsal instrumentation. This strategy was mainly chosen in adjacent-level cases, when direct microsurgical decompression was considered necessary to achieve sufficient release of adhesions and scar tissue before placement of the LLIF cage. In particularly pronounced neuroforaminal stenosis, wide decompression - partially with facetectomy was opted for, which is reflected by the significantly longer duration of surgery and higher blood loss rates. Hence, observed differences in surgery duration and blood loss likely reflect underlying variations in case complexity rather than purely the effect of surgical sequence.

### Surgical approach

Our two-staged approach was designed to maximize deformity correction while minimizing surgical trauma. A matched cohort study comparing OLIF plus posterior fixation to PLIF demonstrated significantly less blood loss, fewer fixed segments, and fewer osteotomies in the OLIF group, with equivalent radiographic correction [11]. These findings support our use of LLIF with posterior fixation as a minimally invasive alternative capable of achieving alignment comparable to that achieved with open surgery. In our series, segmental lordosis correction was most effective in the upper lumbar spine (L1–L3).

The length of stay (LOS) further underscores the advantages of minimally invasive strategies. Kobayashi et al. reported a mean LOS of 20.8 ± 9.8 days in 1,168 patients undergoing predominantly open PLIF/TLIF (mean age 65.9 years) [13]. In contrast, our cohort of similar age (67.7 ± 11.7 years) had a shorter LOS of 13.6 days. Within subgroups, LOS was significantly lower in the LLIF-first group than in patients undergoing LLIF as the second stage, in whom additional decompression and scar release were more common (10.8 vs. 18.9 days, *p* < 0.001).

Hiyama et al. showed that XLIF (indirect decompression) achieved shorter operative times, less blood loss, and superior improvement in back pain compared with MIS-TLIF (direct decompression) [12]. Consistent with these findings, posterior fixation procedures in our LLIF-first group were shorter and less bloody than in the LLIF-second group. Early pain reduction also favored the LLIF-first strategy (ΔNRS: − 2.46 vs. − 1.75), supporting the concept that indirect decompression provides meaningful early benefit, although the difference did not reach statistical significance.

### Segmental lordosis correction by spinal level

In this study, restoration of segmental lordosis (SL) was achieved within previously reported ranges for lateral interbody fusion techniques, demonstrating consistency and efficacy across lumbar levels. We systematically evaluated lordotic changes separately at upper lumbar (L1–L3), mid-lumbar (L3–L4), and lower lumbar (L4–L5/S1) segments. Our results indicated an average segmental lordotic increase of approximately 2°–4° per level, consistent with previously published findings reporting typical corrections below 10°, generally in the range of 2°–4° per treated segment [9, 20]. Specifically, the greatest improvements were documented at the upper lumbar levels, achieving a mean increase of 6.4°.In comparison, mid-lumbar segments showed a modest mean increase of approximately 1°, and the lower lumbar segments exhibited minimal correction (~ 0.3°). This differential correction across lumbar levels aligns with anatomical and biomechanical factors reported previously. Hiyama et al., for example, documented a mean segmental lordosis gain of 3.7° per XLIF-treated level, with notably greater lordosis achieved when cages were placed anteriorly [9]. Similarly, Bakare et al. reported incremental lordotic gains that directly correlated with cage angle, achieving approximately 3° increases with standard (6°) lordotic cages and around 7° increases with more pronounced lordotic (10°) cages [2]. Our study predominantly used standard lordotic cages, yielding comparable corrections, particularly at the upper lumbar levels. The limited correction at the lower lumbar levels (L4–L5/S1) observed in our study is consistent with the established literature, which highlights inherent anatomical constraints at these segments, such as the iliopsoas muscle bulk, facet joint arthropathy, and stiffness that limit extensive lordotic correction solely via lateral interbody techniques [9]. To achieve substantial lordotic restoration at these lower levels, additional surgical maneuvers, such as anterior longitudinal ligament (ALL) release or posterior osteotomies, are typically required—techniques beyond the scope of our protocol. Despite these anatomical limitations and the advanced age of our cohort, the achieved segmental lordosis corrections were clinically meaningful and likely contributed to the observed symptomatic improvement.

### Complication rates in elderly or comorbid patients

An essential aspect for adopting a staged surgical strategy in our study was the reduction of perioperative stress. The cohort analyzed in this study comprised patients with a mean age approaching 70 years and a notable comorbidity burden, as evidenced by elevated CCI scores. The overall complication rate of 25% observed in our cohort appears relatively high and warrants careful interpretation. However, the majority of complications were minor and manageable, including impaired wound healing, transient thigh numbness, and mild medical complications. Notably, there were no occurrences of life-threatening intraoperative events or permanent neurological impairments. ICU stay was short and primarily required for safe postoperative extubation rather than due to cardiorespiratory complications. Our findings are comparable to those documented in earlier large-scale studies—for instance, Spiker et al. reported a 9% complication rate at one year in patients undergoing single-stage XLIF with posterior instrumentation for degenerative spondylolisthesis [18]. Prior literature consistently demonstrates that complication risks in minimally invasive lateral fusion surgery generally increase with patient age and the extent of comorbidities. For example, a large adult spinal deformity series with a mean patient age of approximately 68 years reported a complication rate of approximately 21% for XLIF combined with posterior instrumentation, compared to just 9% when the lateral procedure was performed without posterior fixation [9]. By dividing the surgical intervention into two stages, our approach potentially mitigates the physiological impact of prolonged single-stage procedures. The predominantly minor complications observed in our cohort suggest that staging did not increase surgical risk beyond that reported for single-stage approaches. Significantly, our analysis identified a clear association between higher CCI scores and increased complication risk (OR 2.6 per additional point). This aligns with well-established evidence indicating that greater comorbidity burden correlates with heightened perioperative risk in spinal surgeries [3, 11]. Nonetheless, an important clinical finding of our study is that, despite the elevated complication risk, patients with higher comorbidity indices experienced clinical improvement in pain relief and functional recovery. These results emphasize that carefully selected elderly patients with substantial comorbidities should not necessarily be excluded from surgery, as they can achieve clinical benefits from a thoughtfully planned staged surgical strategy.

### Limitations

One of the study’s limitations is its small sample size: the cohort of 20 patients limits the statistical power to detect subtle subgroup differences and generalizability. Hence, some observed trends—such as differences in pain reduction depending on surgical sequence—did not reach statistical significance and should be interpreted cautiously. The absence of a control group (e.g., ALIF procedures, single-stage fusion or stand-alone LLIF) precludes direct comparisons regarding the relative safety or efficacy of the two-stage approach. The comparison between LLIF-first and posterior-first subgroups is limited by the non-randomized study design and potential baseline differences in pathology severity. Therefore, these findings should be interpreted with caution and primarily considered hypothesis-generating.

Radiographic evaluation of segmental lordosis relied on available imaging. Variability in positioning between pre- and postoperative scans may affect angle precision. Future studies with larger cohorts and standardized radiographic assessment are required to better understand segment-specific alignment changes.

Additionally, while pain scores were collected, functional scores (e.g., ODI) were unavailable. Also, pain assessments were not standardized with respect to timing due to the retrospective design, which may introduce variability in the reported outcomes. The absence of standardized long-term follow-up represents a key limitation of this study. As outcomes were assessed only during the early postoperative period, no conclusions can be drawn regarding the durability of clinical improvement, long-term maintenance of spinal alignment, or delayed complications. Future prospective studies incorporating structured long-term follow-up and patient-reported outcome measures (PROMs) are warranted to better define the sustained clinical and functional benefits of staged lumbar fusion strategies.

Lastly, although comorbidity burden was systematically assessed using the age-adjusted Charlson Comorbidity Index, we did not precisely determine frailty, nutritional status, or psychosocial factors. Despite these limitations, the present study provides meaningful insight into the feasibility and potential advantages of a staged LLIF-posterior instrumentation approach in older, comorbid patients undergoing complex lumbar fusion. In future work, we plan to collect prospective data, including patient-reported outcomes and functional scores, to validate the trends observed in the present study.

## Conclusion

A staged lumbar fusion strategy combining lateral lumbar interbody fusion with posterior instrumentation appears to be a feasible treatment option for elderly and comorbid patients with complex degenerative lumbar disease. The approach was associated with early postoperative pain reduction, segmental alignment changes, and acceptable complication rates.

Our findings suggest that performing LLIF as the first stage may be associated with improved indirect decompression and shorter hospitalization.

Overall, the findings of this study should be considered exploratory given the small sample size. Further studies with larger cohorts, standardized outcome measures, and long-term follow-up, including patient-reported outcomes, are required to better define the role of staged circumferential fusion strategies.

## Data Availability

Retrospective patient data is available from the corresponding author on reasonable request.
